# The impact of programs for prevention of mother-to-child transmission of HIV on health care services and systems in sub-Saharan Africa - A review

**DOI:** 10.1186/s40985-017-0072-5

**Published:** 2017-12-05

**Authors:** Jean Claude Mutabazi, Christina Zarowsky, Helen Trottier

**Affiliations:** 1 Département de Médecine Sociale et Préventive, École de Santé Publique, Université de Montréal, Pavillon 7101, Avenue du Parc, Montreal, QC H3N 1X7 Canada; 2 Centre de Recherche du Centre Hospitalier de l’Université de Montréal (CRCHUM), Tour Saint-Antoine, 3rd Floor, Room: S03.516, 900, Rue St-Denis, Montreal, QC H2X 0A9 Canada; 3 Centre de Recherche du Centre Hospitalier de l’Universitaire Sainte Justine, Montréal, QC H3T 1C5 Canada; 4 School of Public Health, University of the Western Cape, Robert Sobukwe Rd, Bellville, 7535 South Africa

**Keywords:** Maternal and child health, PMTCT, Health systems integration, Sub Saharan Africa

## Abstract

**Background:**

The global scale-up of Prevention of mother-to-child transmission (PMTCT) services is credited for a 52% worldwide decline in new HIV infections among children between 2001 and 2012. However, the epidemic continues to challenge maternal and paediatric HIV control efforts in Sub Saharan Africa (SSA), with repercussions on other health services beyond those directly addressing HIV and AIDS. This systematised narrative review describes the effects of PMTCT programs on other health care services and the implications for improving health systems in SSA as reported in the existing articles and scientific literature. The following objectives framed our review:

1. To describe the effects of PMTCT on health care services and systems in SSA and assess whether the PMTCT has strengthened or weakened health systems in SSA
2. To describe the integration of PMTCT and its extent within broader programs and health systems.

**Methods:**

Articles published in English and French over the period 1st January 2007 (the year of publication of WHO/UNICEF guidelines on global scale-up of the PMTCT) to 31 November 2016 on PMTCT programs in SSA were sought through searches of electronic databases (Medline and Google Scholar). Articles describing the impact (positive and negative effects) of PMTCT on other health care services and those describing its integration in health systems in SSA were eligible for inclusion. We assessed 6223 potential papers, reviewed 225, and included 57.

**Results:**

The majority of selected articles offered arguments for increased health services utilisation, notably of ante-natal care, and some evidence of beneficial synergies between PMTCT programs and other health services especially maternal health care, STI prevention and early childhood immunisation. Positive and negative impact of PMTCT on other health care services and health systems are suggested in thirty-two studies while twenty-five papers recommend more integration and synergies. However, the empirical evidence of impact of PMTCT integration on broader health systems is scarce. Underlying health system challenges such as weak physical and human resource infrastructure and poor working conditions, as well as social and economic barriers to accessing health services, affect both PMTCT and the health services with which PMTCT interacts.

**Conclusions:**

PMTCT services increase to some extent the availability, accessibility and utilisation of antenatal care and services beyond HIV care. Vertical PMTCT programs work, when well-funded and well-managed, despite poorly functioning health systems. The beneficial synergies between PMTCT and other services are widely suggested, but there is a lack of large-scale evidence of this.

## Background

The global scale-up of Prevention of Mother-To-Child Transmission (PMTCT) services is credited for a 52% worldwide decline in new Human Immunodeficiency Virus (HIV) infections among children between 2001 and 2012 [1]. With adequate efforts, more funding and closely monitored progress, the United Nations Programme on HIV and AIDS (UNAIDS) still reported 160,000 new paediatric HIV infections in 2016 [2]. Despite significant progress, the epidemic continues to challenge maternal and paediatric services in Sub Saharan Africa (SSA), with repercussions on other health services beyond those directly addressing HIV and Acquired Immunodeficiency Syndrome (AIDS) [3].

This systematised narrative review describes the impact and integration of PMTCT programs on other health care services. We begin with an overview of prevention of mother-to-child transmission of HIV in the context of global AIDS control efforts, as well as an introduction to the concepts of integration of “vertical” or disease-based programs into broader health systems. We then present and discuss the findings of our review after having presented the methodology.

## Overview: HIV, PMTCT, and health system integration

### Preventing – and eliminating – mother-to-child transmission of HIV

The highest prevalence of HIV infection is in sub-Saharan Africa, where rates of both prevalent and new infections are consistently higher among women than men and most women are now diagnosed during pregnancy or at delivery through antenatal and perinatal care [4]. The Mother-To-Child Transmission (MTCT) of HIV refers to the spread of HIV from an HIV positive woman to her child either during pregnancy, childbirth (labour, delivery) or breastfeeding. MTCT is the most common mode of transmission of HIV to children. Over 90% of children are infected with HIV through MTCT [5]. The interventions aiming to prevent paediatric HIV/AIDS and for better health of both mothers and their children are known as “prevention of mother to child transmission of HIV” or PMTCT [6].

Since MTCT can be prevented through antiretroviral therapy (ART), a global campaign for its virtual elimination was launched on 21 May 2009 as one of the top priorities for the World Health Organisation (WHO), the Joint United Nations Programme on HIV and AIDS (UNAIDS), the United Nations Children’s Fund (UNICEF) and the United Nations Population Fund (UNFPA) [7]. The policy of eliminating new paediatric HIV infections depends on countries reaching not only high rates of initiating ART among pregnant HIV-infected women but also maintaining and supporting them to adhere to treatment [8].

### PMTCT interventions

PMTCT, also called “prevention of vertical transmission”, [9] has been widely implemented in hospital and clinic services particularly in those dealing with antenatal, perinatal and postpartum care. In 2007, WHO and UNICEF developed a guideline document to scale up PMTCT, focusing on resource-constrained settings and efforts towards universal access for women, infants and young children in order to eliminate HIV and AIDS among children [10]. The intervention elements to prevent MTCT are known as the PMTCT cascade [6, 10], outlined in summary form (Table 1).

**Table 1:** PMTCT cascade

PMTCT CASCADE (focusing primarily on components 3 and 4, preventing transmission to infant and treating mother and infant)		
Component	Linked to	
1. Utilisation of antenatal care (ANC)	ANC services	All pregnant women
2. Receiving HIV pretest counselling	ANC unit or voluntary counselling and testing (VCT) services	
3. Acceptance of HIV test	ANC/VCT services	
4. Receiving HIV test results & post-test counselling	ANC/VCT services	
5. Get CD4 assessment	ANC/VCT services	HIV- positive Pregnant women
6. Use of ARV prophylaxis for mom and/or baby (for seropositive moms)	ANC services/ARV programs	
7. Adherence to ARVs during pregnancy	ANC unit/ARV programs	
8. Deliver with skilled attendant & Take ARVs	Obstetric services	
9. Follow safe infant feeding practices	Child health services	
10. Bring infant for HIV testing	Child health or VCT services	
11. Adhere to maternal/infant ARVs after birth	Child health, obstetric or VCT services	
12. Use postpartum family planning	Obstetric services	

This PMTCT cascade reduces the chances for HIV to pass from an HIV-positive mother to her baby during pregnancy, labour, or delivery, or through breastfeeding [5, 6, 11]. Four components of the comprehensive PMTCT programme are articulated by WHO and UNICEF, namely: (1) primary prevention of HIV infection among women of childbearing age; (2) preventing unintended pregnancies among women living with HIV; (3) preventing HIV transmission from a woman living with HIV to her infant; and (4) providing appropriate treatment, care and support to mothers living with HIV and their children and families [6, 10]. The PMTCT cascade is partly or fully implemented by many actors from public and private for-profit and not-for-profit health care sectors (Non-Governmental Organisations (NGOs)), religious and community groups) operating locally but managed and funded at local, national and international levels [12–14].

These multiple actors with their diverse agendas and policies initially delivered PMTCT services as a stand-alone and externally funded programme. The programmes progressively won the interest of governments and are now increasingly supported through public funding in many countries, while still requiring substantial donor support [15]. With time, the strong links this cascade has with maternal and child health services required closer collaboration with and increasing integration into broader services towards sustainable outcomes [14, 16].

The worldwide expanded access to PMTCT services prevented more than 670,000 children from acquiring HIV from 2009 to 2012 [17]. In 2012, over 900,000 pregnant women living with HIV globally accessed PMTCT services - a coverage of 62% - and only 160,000 new paediatric HIV infections were reported in 2016, compared to 300,000 in 2010 [2]. Four African countries (Botswana, Ghana, Namibia and Zambia) had achieved 90% PMTCT coverage [17] while the PMTCT coverage was over 90% and over 80% in Rwanda and South Africa respectively by 2014 [18, 19].

Based on these promising PMTCT figures in SSA, it is possible to envisage achieving virtual elimination of MTCT. In addition, one could expect to see effects of PMTCT programs on other health care services because PMTCT is now largely implemented through existing maternal health programs and services. However, effects on health services and systems are likely to be uneven and complex.

PMTCT is a complex intervention with many involved actors and policies, flows of knowledge, materials, technologies and funds, interacting at global, national and local levels [20, 21]. Beyond the operational challenges of actually delivering the cascade on the ground, this complexity and the history of HIV control programs globally raise systemic and political issues related to the involvement of external funders, experts and manufacturers – sometimes called the global AIDS industry - in funding and implementing HIV services in low and middle-income countries [22]. In addition, PMTCT programs, especially those which aim to be integrated in and help to strengthen health services and systems, seek to address three health goals, each of which is itself a major effort involving different actors, structures and health strategies. These three goals are: combatting HIV/AIDS, reducing child mortality and improving maternal health [12, 23]. Since health services in many countries are organised, funded and managed to deal with distinct diseases and populations but are seen as parts of an overall health system, these multiple interfaces raise questions of whether PMTCT programs have an overall weakening or strengthening impact on national health systems, or no impact at all beyond PMTCT services. For example, Rwanda’s success in scaling up paediatric HIV services through effective utilisation of health resources may offer lessons for other developing countries with high prevalence of maternal and paediatric HIV. This successful integration of PMTCT in Rwanda may be attributed to its health system organisation, despite the weakness of the system [24]. Even so, in contrast to these reported good PMTCT outcomes in countries with a history of stagnating health systems like Rwanda (and most other SSA countries), there is little evidence-informed meaningful discussion about possible PMTCT effects on other maternal and child health services and on overall health systems. This review examines some of these questions.

### PMTCT integration

If we conceptualise PMTCT as a service within a “continuum of care” approach addressing different periods of women’s and children’s life course, PMTCT programs could be seen to have potential beneficial synergies with other reproductive, maternal and child health interventions such as Sexually Transmitted Infection (STI) control, early childhood immunisation, antenatal and delivery care, family planning, nutrition supplementation [16]. For example, maternal deaths have been declining at 2.3% worldwide but this reduction is at only 1.7% in SSA where 9% of all maternal deaths were estimated to be due to HIV/AIDS. The slower rate of decline in maternal deaths in SSA has stimulated interest in a range of recent strategies to increase the coverage of good health care, notably free or very low-cost obstetrical care [25]. The improvement in HIV-related care of pregnant women should help to increase the impact of these other strategies, and thus contribute to accelerated decreases in maternal mortality in high-HIV burden countries.

The potential impact of PMTCT on health services may go beyond maternal and child health specialities and may involve other health care services indirectly, with potential opportunities to enhance overall quality of care but also posing threats such as brain drain and redirection of resources from programs not related to HIV in contexts where there is already weak infrastructure. This raises questions regarding the appropriate approaches to address the challenges regarding the accessibility, equity, and quality of healthcare in the efforts to facilitate service delivery and strengthen health systems [26].

The global monitoring framework and strategy put in place towards elimination of new HIV infections in children [12], reflects this wider perspective of integration across programs and services and calls for comprehensiveness through seven priority areas. These priorities are: (i) Ensure leadership and country ownership; (ii) Improve coverage, access and utilisation of services; (iii) Strengthen quality of Maternal, New born and Child Health services to deliver effective PMTCT of HIV and syphilis interventions; (iv) Enhance provision of linked services; (v) Strengthen human resource capacity, supply chain management and information systems; (vi) Improve measurement of performance and impact and (vi) Develop and engage community systems [12]. This framework does not take PMTCT as a self-sufficient entity but instead calls for seeing PMTCT as integrated within a health system. It thus echoes the WHO-recommended integration of PMTCT programs with other healthcare services to achieve the more accessibility and improvement of health interventions [27]. But what does integration entail, analytically and in practice?

### Health system integration: Vertical, horizontal and diagonal approaches

The 2003 work of Oliveira-Cruz et al. on the synergies between vertical and horizontal health interventions [28], remains relevant today. They define vertical programs as free-standing programs delivering health care services, put in place to deal with a particular disease or condition with predetermined goals and designed for a known time frame and calculated financial means. Horizontal programs refer to service delivery through the infrastructure of the regular healthcare system [28]. While exploring the impact of vertical programs on health systems and experiences of integrating these programs, these authors encouraged the concerted use of both modes of delivery, according to the capacity of a health system as it changes over time [29]. The combination of both delivery models suggests what Julio Frenk and others refer to as a “diagonal approach” [15]. The “diagonal approach” has been referred to as using a disease-specific intervention (e.g. HIV/PMTCT) to strengthen a general health system [15]. Working together across initiatives can bring more benefits; for example, the major investments in vertical programs addressing specific diseases or conditions such as HIV can through diagonal financing cross-subsidise other programs and also the overall functioning of the health system because “horizontal” services like labs and human resources are essential to implement the AIDS, TB and malaria programs [15].

There is no internationally recognised definition of integrated care and to further complicate analysis, some researchers who address what could be considered as integrated service delivery do not use “integration” as a keyword [27]. Integrated care describes a range of organisational arrangements with variable nature and intensity and comprises two main concepts: a) an organisational structure focused on economic benefits, notably efficiency gains, or b) a way of organising service delivery: from no integration, to partial integration or full integration [30, 31]. For both of these approaches, integration is a process that “occurs at different levels of the health system (regional, district, health facility) and in relation to key health system functions namely governance, financing, planning, service delivery, monitoring and evaluation, demand generation” [27, 32]. Analysis of integrated health care requires a good understanding of health care services and health systems components and functions, and integration must be viewed as a process that accommodates complex agendas and issues as was concluded in a study of postnatal care integration into PMTCT in Swaziland [31, 33].

In terms of implementation practices, PMTCT interventions may be carried out in one or more of the following health services: (1) antenatal clinic, (2) delivery/obstetric/labour ward care, (3) postnatal care, (4) neonatal/new born care, (5) paediatric/infant care, (6) nutritional programs, (7) HIV testing and support centres, (8) HIV treatment centres, (9) reproductive/gynaecological services, (10) sexually transmitted infection clinics, (11) family planning, (12) primary health care which in many settings may be largely focused on conditions such as malaria, acute respiratory infection, diarrhoea and malnutrition, (13) Emergency care, (14) Tuberculosis clinics, (15) Malaria clinics (in areas with high burdens of malaria and HIV), (16) Immunisation, (17) or other service [27]. It is worth underlining the close ties between HIV including PMTCT and tuberculosis or/and malaria in terms of coinfection, prevalence, targeted groups and global funding initiatives [34–36]. This variety of potential entry points in the context of the discussion of potential synergies suggests how, and how much, the integration or non-integration of PMTCT could contribute to overall strengthening and integration or weakening and fragmentation of services and systems. In any case, the outcomes depend on how actors collaborate between themselves at health system level, and with the community or individuals seeking health care. A constructive cooperation is encouraged [30, 37] to make integration work and minimise its potential negative effects.

One example that demonstrates the need for cooperative efforts to avoid negative effects of a vertical program on health systems and communities relates to the universal child immunisation goals. A study in six countries in Africa and Asia documented how a top-down model in immunisation interventions ended up creating conflicts between local demand and targets of immunisation policy, leading the authors to argue for more intersectoral collaboration if a specific programme is delivered and managed in a vertical way [28]. This example is one among many that supports calls for shifting from a vertical view of PMTCT programs of HIV prevention and treatment towards a horizontal focus on maternal health care and other health care services [14, 38]. Unfortunately, the lessons that might be learned from immunisation programs and applied to thinking about PMTCT and health systems have not yet been taken fully into account.

Building on this overview of PMTCT and health system integration, this review sought to describe: (1) the effects of PMTCT on health care services and systems in SSA (2) the integration of PMTCT within broader programs and health systems and extent to which it occurred, and a related question (3) whether health systems as a whole have been strengthened or weakened in countries of SSA.

## Methods

### Objectives

This systematically conducted narrative review [39] was based on the following two objectives:

1. To describe the impact of PMTCT on health care services and systems in SSA and assess whether the PMTCT has strengthened or weakened health systems in SSA

## To describe the integration of PMTCT and its extent within broader programs and health systems

### Search strategy

We searched Medline and Google Scholar databases for papers published in English or French between 1st January 2007 (the year of publication of guidelines on global scale-up of the PMTCT of HIV by WHO, UNICEF in partnership with other institutions) and October 2016. We complemented the Medline search with the Google scholar database, looking for any extra articles or grey literature such as policies and programme evaluations important to our review objectives. The following combined search terms were used:

- **Search 1**: (prevention of mother to child transmission or MTCT or PMTCT or (Transmission* or spread*)) adj3 (Mother* adj3 child*)) or HIV or AIDS or acquired immune deficiency syndrome).mp. [mp = title, abstract, original title, name of substance word, subject heading word, keyword heading word, protocol supplementary concept word, rare disease supplementary concept word, unique identifier].
- **Search 2**: (Healthcare or Health care or Primary care or Health systems or Health services or community health systems or Primary health care or Maternal health services or Delivery of care or Health facility).mp. [mp = title, abstract, original title, name of substance word, subject heading word, keyword heading word, protocol supplementary concept word, rare disease supplementary concept word, unique identifier].
- **Search 3**: (sub-Saharan Africa or Africa or Africa south of the Sahara or western africa or eastern africa or central africa or southern africa).mp. [mp = title, abstract, original title, name of substance word, subject heading word, keyword heading word, protocol supplementary concept word, rare disease supplementary concept word, unique identifier].

The identified papers through these three combined searches were all exported to Endnote bibliographic software [40].

The search strategy did not explicitly require the terms “impact” and “integration” because we felt that some studies might address “impact” and “integration” indirectly, through services description. We defined impact based on the existence of one or more areas of public health action framework as demonstrated through the five aspects of Frieden’s 2010 pyramid that underlies intervention with potential health impact [41]. These five aspects are: 1) socioeconomic factors, 2) changing the context to make individual’s default decision healthy, 3) long lasting protective interventions, 4) clinical care, 5) counselling and education [41]. Integration was defined based on the concept of integrated care as it was above explained.

### Inclusion and exclusion

We designed the review inclusion and exclusion criteria for the purpose of retaining all studies pertaining to comprehensive PMTCT components throughout health systems in SSA as discussed in the WHO PMTCT strategy [6]. We looked for the papers that examined this strategy in terms of PMTCT’s positive and negative effects on other health care services or vice versa, and integration of PMTCT programs with other health care services in the paper’s title, results and discussion.

The abstracts of pertinent papers were then retrieved following these inclusion criteria before selecting the full articles: (i) *Papers* - Research articles published in peer-reviewed scientific journals, grey literature and commentaries dealing with PMTCT in pregnant women in SSA were accepted for inclusion. (ii) *Participants* - Women at risk of transmitting HIV infection to their children. This could include pregnant women or those at risk of pregnancy and their children, regardless of HIV status. (iii) *Interventions* - All interventions to prevent or reduce HIV MTCT, including but not limited to strategies for antiretroviral therapy and replacement feeding. PMTCT collaboration with other health care services especially maternal and child health (MCH) were included. The following types of articles were excluded: (i) Studies focusing on countries other than SSA countries, (ii) Studies focusing on general HIV/AIDS prevention or other health care services without reference to PMTCT and (iii) Editorials or commentaries generally describing the PMTCT programs on one or more pre-specified healthcare services without studying its effects or integration.

### Data extraction and analysis

All three reviewers agreed on search strategy and inclusion and exclusion criteria. The initial database was created from the compiled electronic searches by one reviewer. All citations were firstly screened by title and abstract and duplicate citations were eliminated. The full texts of potentially eligible papers were then independently obtained for further screening. After resolving differences in data extraction or interpretation through consensual discussions based on the above stated inclusion and exclusion criteria, the final retrieval of papers was conducted. The following study characteristics and data were extracted from included papers: authors, year of publication and country of study, study types or designs, paper’s focus interface with PMTCT, brief topic of investigation and main results. The findings were organised according to the objectives and referred to as ‘themes’ pertaining to each of two review objectives.

Throughout the whole selection process, the impact of PMTCT programs on other health care services or vice versa was reported in order to describe to how and to which extent it occurred. Integration of PMTCT programs with other health care services at different levels was also described across all included papers. Two guidelines on global scale-up of the PMTCT were also retrieved.

## Results

**Fig. 1:**
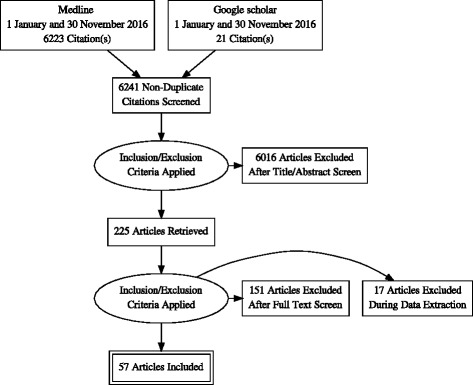
PRISMA flow diagram of articles included in the review

We identified 6223 citations and ultimately retained 57 articles, in addition to the PMTCT guidelines mentioned above. These two guidelines are: 1) The global plan for elimination of new HIV infections among children by 2015 and keeping their mothers alive, and 2) The PMTCT strategic vision 2010–2015: preventing mother-to-child transmission of HIV to reach the United Nations General Assembly Special Session (UNGASS) and Millennium Development Goals [6, 42, 43]. The study selection process is presented in the form of an adapted PRISMA flow-diagram (Fig. 1) and the retained articles are summarised in Table 2.

**Table 2:** Characteristics of included articles

No. Autors, year and country of study	Study types or designs	Focus interface with PMTCT	Brief topic of investigation	Main results
1. Bancheno WM et al. 2010/ Swaziland [45]	Mixed	Maternal and child health (MCH)	Reporting the outcomes and challenges of a comprehensive service to PMTCT	Provision of a complete package of PMTCT services in resource limited setting is possible but challenged by staff shortage, socio-economic and service-related factors
2. Barker PM et al. 2011/ RSA [46]	Quantitative	Health systems	The impact of health systems’ performance on MTCT	It’s necessary to support PMTCT interventions at scale to achieve gains in maternal and child survival
3. Barker PM; Mate K. 2012/ Multicountry [47]	Quantitative	MCH	Eliminating mother-to-child HIV transmission will require major improvements in maternal and child health services	Access to maternal and child health services along with prevention measures and HIV treatment before pregnancy need to dramatically be improved
4. Barry OM et al. 2012/ RSA [48]	Quantitative	Antenatal care (ANC)	The patient-provider relationship (PPR) in antenatal care and its importance in PMTCT	PPR scale is useful and context-appropriate instrument that could have an important role in future research focusing on improved PMTCT and decreasing the risk of paediatric HIV
5. Behets F et al. 2009/ RDC [49]	Quantitative	ANC, maternal health	Reducing vertical HIV transmission	Initiating vertical HIV transmission prevention embedded in improved antenatal services in a fragile, fragmented, severely resource-deprived health care system was possible and improved over time
6. Both JMC et al. 2010/ Multicountry [14]	Systematic review	Maternal health	The impact of Prevention of PMTCT programs on maternal health care (MHC)	Integrated PMTCT programs can lead to more positive effects despite its current positive and negative impact on MHC
7. Chi BH et al. 2013/ Multicountry [50]	Review	Health facilities, health policy	Antiretroviral drug regimens to prevent mother-to-child transmission of HIV	Global campaigns to “virtually eliminate” paediatric HIV and dramatically reduce HIV-related maternal mortality have mobilised new resources and renewed political will
8. Cotton MF et al. 2008/ RSA [51]	Quantitative	Tuberculosis (TB)	Tuberculosis exposure in HIV-exposed infants in a high-prevalence setting	Programs to prevent MTCT offer an important opportunity to screen for TB. In-depth assessment is required for evaluating TB exposure.
9. DeGennaro V; Zeitz P. 2009/ Multicountry [52]	Review	Family, paediatric AIDS	Family-centred response to the HIV/AIDS epidemic for the elimination of paediatric AIDS	Governments must embrace family-centred and implement paediatric-friendly infrastructure, and train healthcare workers to treat children care in order to eliminate paediatric HIV/AIDS
10. Desclaux A et al. 2012/ Senegal [75]	Qualitative	Social issues	Social stakes to eliminate MTCT by 2015	Integration of HIV testing and treatment in RH services is not fully established and health services organisation hinders family approach of prevention
11. Druce N; Nolan A. 2007/ Multicountry [44]	Review	Maternal health	linking HIV and maternity care services	Experience in some countries has shown that progress can be made whenever nationals are committed to resources increase to meet HIV maternal and newborn needs
12. Du Plessis E et al. 2014/ Kenya [53]	Quantitative	ANC	Challenges to PMTCT implementation	Guideline implementation in low resource settings continues to be confronted with challenges related to late presentation, less than four ANC visits, low screening rates for opportunistic infections and limited contraception counselling
13. Ekouevi DK et al. 2012/ Multicountry [54]	Mixed	Health facilities	Health facility characteristics associated with effective PMTCT	There is a positive relationship between an antenatal quality score and PMTCT coverage but variables to predict PMTCT coverage were not identified
14. Evjen-Olsen B et al. 2009/ Tanzania [76]	Descriptive*	MCH	Integrating PMTCT on healthcare services	Horizontal and comprehensive services should be strengthened and supported at all levels towards a sustainable MCH impact
15. Ferguson L et al. 2012/ Multicountry [77]	Systematic review	Maternal health, ANC and post-natal care (PNC)	Linking women who test HIV-positive in pregnancy-related services to long-term HIV care and treatment services	Providing ‘family-focused care’, integrating CD4 testing and providing HAART into PMTCT services are promising for increasing women’s uptake of HIV related services
16. Gibbs A et al. 2012/ Multicountry [55]	Analytical**	MCH	Inclusion of women, girls and gender equality in National Strategic Plans for HIV and AIDS	There is poor inclusion, except the accessible post-exposure prophylaxis in the case of sexual violence and vertical transmission services
17. Govender T; Coovadia H. 2014/ Multicountry [56]	Review	ANC, MCH	Eliminating mother to child transmission of HIV-1 and keeping mothers alive	Family planning messages and provision of contraception methods to avoid unplanned pregnancies are more effective than HIV counselling and testing, and single dose Nevirapine in averting transmission of perinatal HIV infection.
18. Gunn JK et al. 2016/ Multicountry [98]	Quantitative	ANC	Relationship between ANC and PMTCT in SSA. Analysis of data from four countries.	Integration of HIV testing into routine ANC service is needed to increase opportunities for PMTCT programs to reach HIV-positive pregnant women.
19. Harrington EK et al. 2012/ Kenya [78]	Quantitative	Family planning (FP)	Fertility intentions and interest in integrated FP services among women living with HIV	Integration of FP and HIV services is acceptable and should be supported for preventing the unintended pregnancies through increased access to contraceptive methods and confidential services that take into account women’s varied reproductive intentions
20. Hatcher AM et al. 2014/ RSA [57]	Qualitative	Partners of PMTCT women	Links between HIV and intimate partner violence in pregnancy	HIV diagnosis during pregnancy and subsequent partner disclosure are common trigger of IPV
21. Hoke T et al. 2014/ RSA [79]	Mixed	FP	Contraceptive options for PMTCT clients	Consistent access to high quality FP services that are effectively linked to HIV services, attention must also be focused on resolving underlying health system constraints weakening health service delivery more generally.
22. Horwood C et al. 2010/ RSA [80]	Mixed	MCH	Evaluating PMTCT implementation and integration into routine maternal, child and women’s health services	Poor integration of PMTCT services into routine care, lack of clarity about health worker roles and poor record keeping constitute barriers to services accessibility in post-partum
23. Jashi M. et al. 2013/ Multicountry [81]	Review	Paediatric HIV care	PMTCT - paediatric HIV joint technical missions	Joint technical missions bring together stakeholders for common action towards informed PMTCT and paediatric HIV policies
24. Kaida A et al. 2010/ RSA [82]	Quantitative	Contraceptives	Investigating the contraceptive use and method preferences by HIV status and receipt of HAART among women	Integrated HIV and sexual and reproductive health services have potential to positively impact maternal, partner, and child health.
25. Karl Peltzer et al. 2009/ Multicountry [58]	Randomised control trial (RCT)	FP	Family Planning - effects of PMTCT	HIV prevention and FP should consider the reproductive desires of HIV positive women and men
26. Kerber KJ et al. 2013/ RSA [83]	Systematic review	Child deaths	Meeting Millennium Development Goal 4 through HIV services	Failing to address other aspects of care like the integration of high-quality maternal and neonatal care leads to low decline in child mortality
27. Kerouedan D. 2010/ Multicountry [59]	Evaluative***	Global Fund- health policy and systems	Evaluating policy issues	Health systems’ weaknesses at district level, such as human resources, laboratory commodities, and medicine shortages are major constraints to further expansion of services and impact of funds
28. Ladur AN et al. 2015/ RSA [60]	Qualitative	Male involvement	Perceptions on Male Involvement in PMTCT	HIV testing, disclosure and direct health worker engagement with men increases male involvement in PMTCT.
29. Lassi ZS et al. 2014/Multicountry [61]	Review	Neonatal health	Essential pre-pregnancy and pregnancy interventions	Proper implementation of a set of essential interventions leads to both improved and sustained maternal, neonatal and child health outcomes
30. Levy JM. 2009/ Malawi [62]	Qualitative	ANC, MCH	Examining women’s decisions about HIV testing and their experience of PMTCT and HIV-related care	Women’s own health was particularly marginalised within the PMTCT programs but good models exist for comprehensive care for women, infants and their families
31. Lyons C et al. 2012/ Multicountry [84]	Commentary & supplement article	MCH	Ending paediatric AIDS and achieving a generation born HIV-free	Scale-up of PMTCT has provided a foundation for HIV prevention and care and treatment programs that are integrated within maternal and child health services
32. Nassali M et al. 2009/ Uganda [63]	Qualitative	MCH, PNC	Adherence to the postnatal PMTCT program	Strategies to increase mothers’ adherence to PN-PMTCT interventions leads to increased HIV/AIDS care access for mothers and children in SSA are recommended
33. Nutman S et al. 2013/ Multicountry [16]	Systematic review	STIs and child immunisation	Assessment of PMTCT impact in all low and middle income countries	There are beneficial synergies between PMTCT programs and both STI prevention and early childhood immunisation.
34. O’Reilly KR et al. 2013/ Multicountry [85]	Systematic review	FP and counselling	FP counselling for women living with HIV	There is a need for strengthened efforts to integrate family planning counselling and access to services into HIV prevention, and for greater consistency of effort over time.
35. Peltzer K et al. 2010/ RSA [86]	Mixed	Health systems	Assessment of the PMTCT implementation across health facilities	A well-functioning health system empowers PMTCT clients through strong leadership, coordination and collaboration between partners.
36. Pirkle CM et al. 2014/ Multicountry [64]	Quantitative	Nutrition	Training and nutritional components of PMTCT programs	Health professionals’ training in maternal healthcare and PMTCT could be combined to improve the quality of obstetric care in the region.
37. Potter D. et al. 2008/ Zambia [65]	Quasi-experimental	MCH, Primary health care	Improve overall care for pregnant women	Broader primary care impact and full PMTCT integration should be targeted.
38. Rollins N et al. 2007/ RSA [87]	Quantitative	Immunisation	Surveillance of MTCT prevention programs at immunisation clinics	Anonymous HIV prevalence screening of all infants at immunisation clinics is feasible and could help to monitor the impact of PMTCT programs in peripartum infection, in identifying the infected children early for referral into care and treatment
39. Roxby AC et al. 2014/ Multicountry [66]	Review	MCH	A lifecycle approach to HIV prevention in African women and children	The potential for synergistic and additive benefits of lifecycle interventions should be considered when scaling up HIV prevention efforts in SSA
40. Ruton H. et al. 2012/ Rwanda [67]	Mixed	Child health, community	PMTCT - community-based household survey	National PMTCT programs in SSA should assess the effectiveness of their interventions to achieve the MTCT elimination goals
41. Rutta E. et al. 2008/ Tanzania [88]	RCT	MCH	PMTCT in a refugee camp setting in Tanzania	Integrated PMTCT (into existing ANC) is more successful and acceptable
42. Sarnquist CC et al. 2013/ Multicountry [89]	Review	SRH and FP	Sexual and reproductive health and family planning needs among HIV-infected women in Sub-Saharan Africa	Integrated services that help prevent unintended pregnancy and optimise maternal and infant health before, during and after pregnancy are also useful for both resources maximisation and improvement of reproductive outcomes
43. Shetty AK. 2013/ Multicountry [68]	Review	MCH, PNC	Epidemiology of HIV infection in women and children: a global perspective	Rapidly implemented combination of HIV prevention packages provides quality PMTC services and improves maternal and infant survival.
44. Spangler SA et al. 2014/ Kenya [69]	Quantitative	Maternal care	HIV-positive status disclosure as factor for the use of PMTCT and maternal care	Interventions to promote HIV disclosure must recognise the impact of HIV-related stigma on disclosure decisions and protect women’s rights, autonomy, and safety.
45. Sprague C et al. 2011/ RSA [70]	Qualitative	MCH	Women’s experiences on continuum of maternal and child care	There are missed opportunities for HIV testing in antenatal care due to huge operational weaknesses in HIV services.
46. Sinunu MA et al. 2014/ Malawi [71]	Quantitative	Immunisation	Immunisation clinic-based surveillance of PMTCT and evaluation of PMTCT impact	Successfully implemented PMTCT program averts HIV transmission and can be evaluated over time for impact through immunisation settings.
47. Tomlinson M et al. 2014/ RSA [90]	RCT	Health systems, community and MCH	Evaluate an integrated, scalable package of pregnancy and post-natal home visits	Implementation of a pro-poor integrated PMTCT and maternal, neonatal and child health home visiting model is feasible and effective.
48. Towle M; Lende DH. 2008/ Lesotho [72]	Qualitative	Community	Social groups and contextual determinants impacting PMTCT	Biomedical system, women, children and the community have to be considered as valuable partners in achieving positive health outcomes.
49. Uwimana J et al. 2012/ RSA [26]	Mixed	NGOs, Community care Workers (CCWs) and TB/HIV	Engagement of NGOs and community care workers in collaborative TB/HIV activities including PMTCT	Efforts to engage the NGO/CCWs for implementing collaborative TB/HIV/PMTCT activities are beneficial but sub-optimal in practice.
50. Uwimana J et al. 2012/ RSA [91]	Qualitative	TB/HIV	Health system barriers to implementation of collaborative TB and HIV activities including PMTCT	Accelerated implementation of collaborative TB/HIV activities including PMTCT requires political will and leadership to address these health systems barriers.
51. Uwimana J et al. 2012/ RSA [92]	RCT	TB/HIV	Training community care workers to provide comprehensive TB/HIV/PMTCT integrated care	Up-skilling CCWs could be one avenue to enhance TB/HIV case finding, TB contact tracing and linkages to care.
52. Uwimana J; Jackson D. 2013/ RSA [93]	Qualitative	TB	Assessing the integration of TB services into the PMTCT	The inadequate integration of TB prevention and care into the PMTCT programme will require a strong leadership that addresses training and supervision barriers.
53. Uwimana J et al. 2013/ RSA [94]	RCT	TB-HIV	Impact assessment of an intervention to enhance the provision of community-based integrated services for TB, HIV and PMTCT	The effective intervention in enhancing the provision of community-based TB-HIV and PMTCT services requires more attention to other primary health care services to ensure that all key services are provided.
54. Vermund SH; Hayes RJ. 2013/ Multicountry [95]	Review	MCH, PNC	Combination prevention to stop HIV	Combination approaches are complex and costly. They require substantial global commitments.
55. Wettstein C et al. 2012/ RSA [96]	Systematic review	MCH	Determining the magnitude and reasons of loss to program and poor antiretroviral prophylaxis coverage in PMTCT	Uptake of PMTCT interventions and early infant diagnosis is unsatisfactory. An integrated family-centred approach seems to improve retention.
56. Wiysonge CS et al. 2011/ Multicountry [73]	Review	ANC, PNC, Vitamin A	Vitamin A supplementation for reducing the risk of MTCT	vitamin A supplementation probably has little or no effect on MTCT in antenatal care or in postpartum.
57. Woldesenbet S et al. 2015/ RSA [97]	Mixed	MCH, Communities and health facilities	Risk factors for low PMTCT service uptake	Strengthened linkages of referral-system and between communities and health facilities can address factors to low PMTCT service uptake.

*The paper described a health program in presentation form

**National strategic plans in 20 countries were assessed in this study

***The paper evaluated policy issues and commented on them

Table 2 describes the included studies in which different types of research designs and data collection methods were used. Two papers were categorised as policy-evaluative (one that evaluates PMTCT policies and one that assesses various national strategic plans in 20 countries in Eastern and Southern Africa). One descriptive study presented a health program. Eighteen reviews, nine qualitative studies, thirteen quantitative studies, and eight mixed methods studies were selected along with six controlled trials and one quasi-experimental study. In most of the studies, hospital and clinical records were reviewed and individual or group interviews with women and staff members involved in health care and PMTCT services were conducted by the researchers among the selected areas/sites and participants. Key informants such as policy makers, district health workers, academicians, implementing partners and persons living with HIV were also contacted in the research process in most of selected articles. Many programs and services were classified as fully vertical while a few others were perceived as either semi or fully integrated based on the extent of verticality or horizontality of PMTCT programs [14, 44].

Thirty-two articles recorded the positive and negative effects of PMTCT on other health care services as a major theme in the article’s title and/or discussion, out of fifty-seven [14, 16, 45–74]. Twenty-five articles examined PMTCT and its integration in health systems in SSA as a main theme [26, 44, 75–97]. Twenty-three papers out of 57 were classified as multinational as they covered two or more SSA countries [16, 44, 47, 52, 54–56, 59, 61, 64, 66, 68, 73, 74, 77, 81, 84, 85, 89, 95, 98], twenty examined our research themes in the Republic of South Africa (RSA) [26, 46, 48, 51, 57, 60, 70, 79, 80, 82, 83, 86, 87, 90–94, 96, 97], three in Kenya [53, 69, 78], two each for Malawi [62, 71] and Tanzania [76, 88] and one for each of Democratic Republic of Congo [49], Lesotho [72], Rwanda [67], Senegal [75], Swaziland [45], Uganda [63] and Zambia [65].

Integration as a theme was found in twelve papers on PMTCT integration in ANC, postnatal care (PNC) and child care [44, 76, 77, 80, 83, 86, 88, 90, 95–98], five papers examining PMTCT and HIV services integration with tuberculosis screening and treatment [26, 91–94], six articles on integrating sexual and reproductive health (SRH) and family planning (FP) services to prevent unintended pregnancy and optimise maternal and infant health in ANC and PNC [75, 78, 79, 82, 85, 89], one article on linking immunisation to HIV screening among children [87] and one on paediatric HIV [84]. We found literature examining both impact and integration of PMTCT programs as part of maternal and child health (MCH) services. MCH in this review covers antenatal, delivery and post-partum or post-natal care for both the mother and child. Other elements related to direct MCH services were included, notably social factors such as women’s perceptions, community and male involvements in mother and child services, intimate partner violence and gender equality. The above-mentioned MCH components appeared in a total of twenty-one articles [14, 45, 47–49, 53–55, 57, 59, 60, 62, 63, 65–70, 72, 74].

## Impact of PMTCT on other health care services

PMTCT has been promoted by WHO as a reliable solution to paediatric HIV. As a comprehensive approach developed based on the four components, the scaling up of PMTCT became a cornerstone of countries’ HIV prevention, care and treatment programs [42]. As mentioned above, 32 of the 57 retained articles addressed the impact of PMTCT on other health care services. The major findings are the potential improvement of existing MCH services and to some extent increasing the availability, accessibility and utilisation of other PMTCT-related services such as prevention of unintended pregnancies, control of STI, immunisation, nutrition and vitamins supplementation [16, 44, 73, 76, 78, 87, 89]. PMTCT services also offer, to some extent, opportunities for screening tuberculosis among exposed infants [51] and may generally enhance diagnosis and treatment through collaboration efforts at both health care settings and community [26, 91, 92, 94].

Several issues surrounding the PMTCT roll-out are reported to adversely impact PMTCT outcomes [14] and these include its process, services delivery, quality of supplies and tools, hiring and training of adequate staff, among others. However, conclusions regarding the impact of PMTCT must be cautious due to lack of detailed studies analysing such effects. In their attempt to quantify the impact of PMTCT programs on overall health systems in SSA, Nutman et al. [16] specifically assessed the existing knowledge and evaluated the PMTCT services impact beyond HIV transmission prevention. They also looked at how these programs contribute to the broader health outcomes. In the end, their systematic review of literature published up to 2011 found evidence of numerous beneficial synergies with specific health services but that there was insufficient evidence to draw any firm conclusions about the broader impacts of PMTCT on health outcomes or health systems. They report serious gaps regarding appropriate recording, data availability and information flow, and argue that these gaps might mislead decision-making, funding allocation and implementation of initiatives.

## Health system strengthening or weakening

Health systems link people with promotive, preventive, curative, rehabilitative or palliative health services to address health problems. The effectiveness of this linkage depends on many factors, including those outside the health sector as well as within the various components of a health system [99, 100]. We identified six articles explicitly or indirectly addressing the broader health system [44, 46, 59, 86, 90, 91] while the remaining fifty-one focused internally, on one or more components of the building blocks and functioning of health systems and services.

Health care providers and funders across SSA have shown interest and engagement to make PMTCT services more and easily accessible to women and children [16, 47, 84, 101] but their efforts have not eliminated many challenges underlying SSA health systems. Added to social problems, these health system issues create a situation in which it is difficult for women to actually utilise services during pregnancy and postpartum, even when these are offered free of charge [76, 102, 103]. Looking within the health systems themselves, PMTCT services have positively impacted MCH and adequately reduced the spread of HIV infection [16], but optimal outcomes occur where the health services are delivered in conducive working conditions, with adequately equipped facilities and committed management [104]. Such conditions are rare in SSA where the vertical transmission of HIV is the highest in the world. PMTCT and other HIV services in SSA depend on foreign funds estimated at billions of dollars but unfortunately the overall system and population-level results of such efforts and investments are seen as mixed [21].

Studies in this review pointed to these shortcomings and deficiencies in service delivery, especially for the components regarding the uptake of PMTCT programs such as antenatal HIV testing and receipt of test results, ARV prophylaxis and postnatal mother-infant follow up [105, 106]. Campbell et al. [21] therefore argue for a strategy for (i) ‘translating’ intervention approaches into locally and culturally appropriate discourses and practices; (ii) building local capacity to sustain interventions once their funded period is over; and (iii) strengthening health systems in affected settings.

Analysing the effects of PMTCT on other health care services is often framed in terms of a debate over whether the roll-out of HIV/AIDS services including PTMTC did or did not strengthen the existing health systems and services. Studies in our review demonstrated the advantages of scaling up HIV services in terms of saving many lives, training health providers, and funding some key services like MCH, but the same findings highlighted the various shortcomings of HIV/AIDS interventions in health systems of low and middle-income countries and particularly in SSA [14, 27, 44, 65, 102, 106]. Some encouraging results suggest positive impact of PMTCT programs on health systems [44] but also highlight the challenges that PMTCT and health systems did not meet; these unmet challenges are seen as negative effects mostly caused by poorly resourced settings facing a high disease burden [14]. These challenges include among others: continued high rates of home deliveries, shortages of personnel, inadequate supplies of test kits, varying distribution and availability of PMTCT service delivery points, lack of supplementary feeds for women who may opt for non-breast feeding for their infants, and logistical and social implications after testing HIV positive, such as a lack of spousal support and sometimes violence [14, 103, 106, 107]. None of the retained papers showed or argued that PMTCT programs directly weaken health systems. In order to strengthen health systems, the implementation of PMTCT and disease-specific interventions needs more collaborative efforts to address structural, organisational, managerial and finance barriers.

## Integration of PMTCT within broader programs and health systems

Even though some programs remain disease-specific, our review found that PMTCT programs and other health services mutually interact. PMTCT integration within general health care services in low and middle income countries has also been recommended to boost the utilisation of these interventions [27]. Some studies noted that under a PMTCT umbrella, the quality of other services in integration is also being closely monitored and improved [102, 105].

PMTCT integration has for example, positively influenced maternal and child care services regarding service availability, accessibility and utilisation [44]. Evjen-Olsen et al. [76] suggested that maternal and neonatal health can be improved by integrating health care services, supporting integration of health systems rather than separately organising and managing different vertical and horizontal programs especially in developing countries [108]. The articles reviewed here also identified potential synergies between the integration or combination of PMTCT with specific health care activities outside of direct obstetrical and child care or MCH services, including sexually transmitted infection (STI) control and immunisation [16, 71], sexual and reproductive health and family planning [56, 58, 61], nutrition [64], tuberculosis [51] and vitamin A supplementation [73]. The synergies are variously achieved in different contexts through progressive efforts, such as staff training and motivation, planning and evaluation of services, restructured management and financing among others. In terms of integration itself as a theme, the following synergies were examined by retained studies, apart from MCH: HIV/PMTCT services integration with tuberculosis screening and treatment [26, 91–94], with SRH, STIs and FP services [16, 75, 78, 79, 82, 85, 89] and with immunisation and HIV screening among children [87]. These linkages helped to increase and improve training of care providers, to review and enhance funding and implementation policies, to increase access and adherence to services, to reduce drug stock outs and improve basic infrastructures.

While identifying and describing the effects of PMTCT on other health services and health systems or the integration of PMTCT and its extent within broader programs and health systems, collaborations and involvements at different levels emerged to be crucial. Since health services are not only provided by the public sector, our search included other organisations offering PMTCT services and other actors involved in offering such services outside of the public sector. We identified examples of service delivery provided by NGOs [26], or delivered in refugee camps [88] while one paper focused on male partner involvement [60]. While studies examining public health services tended to focus on activities within clinics and services, these additional papers reveal that PMTCT activities shape interactions between the community members, social organisations and clinics offering the PMTCT services. The collaborations reported in some articles were directed to implementation or evaluation of initiatives against stigma, for the “normalisation” of HIV as just another other disease and to increase the accessibility of other social and support services to women living with HIV [6]. Selected articles stressed collaboration initiatives so that the PMTCT programs could become a model in “networking, nurturing relationships and bringing all available resources and agents to the table to find solutions and forge partnerships in order to procure all elements essential to a high quality, comprehensive, integrated program” [42].

## Discussion

The review aimed to document the possible effects of PMTCT on health care services and systems and the integration of PMTCT within broader programs and health systems in the available literature in SSA. The analysis of available evidence regarding the two main aspects of this review, namely PMTCT impact and integration, supports a generally positive evaluation of positive synergies with MCH as well as other health services, suggests increasing partial integration of PMTCT within health systems, and offers inconclusive arguments on whether health systems as a whole have been strengthened or weakened by the PMTCT programs in countries of SSA. The discussion below addresses three issues that must be further studied and analysed in order to increase the likelihood that health systems will be demonstrably strengthened: the availability and quality of information, synergies and impacts within and beyond the health sector and the need to engage programmes and services beyond MCH, sexual and reproductive health, and TB.

Beyond the structural limitations on the positive impact that PMTCT programs might have on health systems in SSA, there is also a crucial continuing problem of accurate and timely information as highlighted by Theuring et al., (22), and a lack of rigorous research regarding the effects of PMTCT programs on health care systems or vice versa. While all included papers contributed to one or another dimension of the research questions, only two among 32 retained articles that recorded the impact of PMTCT, explicitly and directly focused on or addressed the impact of PMTCT on other health services and on health systems [14, 16]. This is a very unusual scenario, considering the large body of available literature on HIV/AIDS interventions in SSA. It is worth repeating that Nutman et al. [16] specifically aimed to evaluate the impact of PMTCT services beyond transmission prevention and assessed the existing knowledge about such programs and how they contribute to broader health outcomes. Unfortunately, these research efforts were stymied by the weakness of health information systems, unreliable administrative and research data, and important evidence gaps. This research gap, coupled with under- or improper reporting practices, poses serious challenges to informed decision making, proper funding allocation and effective implementation.

Likewise, in their 2011 Cochrane review, Lorainne Tudor Car and colleagues did not find any study that evaluated the PMTCT interventions integration with other health care services to improve health outcomes [27]. In another study conducted in Swaziland, a country with a background of high, but stable HIV prevalence, an intervention with triangulated data of HIV testing and counselling, ART, PMTCT, and TB screening programs improved coverage and documented promising health outcomes. Even though the scale-up of this intervention was successful, the lack of data remained a non-negligible challenge throughout the study [109]. A situation like this is not an exception in SSA where health records are often incomplete or poorly managed. Without data storage and sharing, the combination of health interventions becomes more complicated and even impossible. This tendency can only be changed at national and regional levels through viable, coordinated investment and practice to monitor and to improve effects of major programs like PMTCT on other services and health systems or vice versa.

## Synergies, collaborations and impacts on health systems: Positive contributions to a problem beyond the scope of PMTCT and the health sector

The included papers provided moderate evidence of mutual benefits between the PMTCT services and the existing health care services and they recommended a close relationship and more integration in order to maximise the advantages of working together and to mitigate some of the challenges of controlling HIV, a lifelong condition, in the conditions facing SSA [8, 14, 27]. The available scientific studies and reports suggest the existence of both positive and negative effects generated by PMTCT on the maternal health services where they are mostly based and on child health services. The PMTCT services increase not only the accessibility but also the utilisation of antenatal and other MCH services [110]. In addition, the increased access to HIV testing and treatment are transforming HIV into a chronic illness. However, the lifelong management of HIV requires continued commitment and engagement of different actors such as the funders, the health care system, the patients, their families and as well as the community [4]. The efforts to manage the HIV pandemic as a chronic disease and treat the opportunistic infections or any other HIV associated conditions overwhelm SSA’s struggling health care systems. The systems that depend on conditional foreign aid do not enjoy managerial autonomy and cannot consistently plan for their future interventions. PMTCT services within such systems cannot, on their own, strengthen health systems.

As seen in the results section, the failure of PMTCT to demonstrably strengthen health systems is associated and sustained by the underfunded and poorly managed health care settings [14]. HIV counselling and testing is not yet universal and many pregnant women come late to seek prenatal care and they then access all the services at the same time instead of progressing through the whole cascade. Lack of adequate resources and facilities (infrastructure, materials, and human resources among others) are major barriers to successful interventions in SSA where the operational and implementation problems arise from local contextual issues as well as the underlying poverty reflecting global economic forces.

The reality of plural health systems and multiple actors must also be addressed in research, policy, and practice. In SSA, the private for-profit sector and charitable and faith-based health care providers including mission hospitals and NGOs play an important role [26, 91] and should be included in research and policy dialogue. This entails the widely advocated but in practice difficult coordination between well-funded international organisations, struggling national public health systems, NGOs and people living with HIV in search of treatment [111].

Beyond general statements of goals of collaboration and consideration of intervention context, there should be a clear strategy to strengthen local responses to HIV which are often ignored by the top–down style adopted in order to comply with requirements of “the global funding architecture” [112]. This responsiveness to local conditions and actors takes time and requires flexibility. In contrast, the “emergency” nature of much HIV intervention combined with accountability requirements to funders tend to align with a more directive and hence less responsive and collaborative approach. Constructive collaboration is not only advised but critically needed in order to achieve both PMTCT and health services goals. This concerns all models of health care delivery, whether vertical, horizontal or diagonal.

## Beyond MCH, sexual and reproductive health and TB

Malaria, severe anaemia, diarrheal diseases and acute respiratory diseases are some of the leading causes of death among women and children in many of SSA countries [14, 113] including RSA, the source of most studies identified for this review. These are rarely, if at all addressed in PMTCT programs and their impacts on PMTCT or vice versa are not discussed in any study included in this review. This is possibly due to HIV interventions and research continuing to be seen as a separate issue from health conditions other than those directly related to sexual and reproductive health and TB.

In the cited systematic review of Lorainne Tudor Car et al. [27] on integrating PMTCT programs with other health services for preventing HIV infection and improving HIV outcomes in developing countries, the authors decided not to make any recommendation about the implementation of integrated PMTCT programs based on the fact that only one study met their selection criteria. It is logical to conceive and integrate the PMTCT programs within MCH services given the correlation of both services delivery but other services such as SRH or immunisation clinics as the point of entry to PMTCT programs need to be explored. In addition to publications for this review that recommend more research on the impact of PMTCT programs on health care services, the WHO, UNAIDS, UNICEF, UNFPA and PEPFAR calls to integrate the PMTCT and other disease stand-alone programs with other health care services [6, 10, 12, 42] remain relevant.

## Limitations

This a comprehensive and complex review that included a range of issues related to PMTCT impact and its integration on other health services and systems. Even though it was rigorously conducted, not all PMTCT implementation details could be reviewed in this single paper.

The regional HIV prevalence and other crucial factors, such as relationships with donors and international researchers as well as publication outputs, are highly variable across countries and regions in SSA. Most of the identified studies covered eastern and southern Africa. Central and western parts of Africa are less covered in publications, a situation that makes it difficult to generalise this review’s findings to those regions. Studies reporting good service delivery and adherence may also not reflect the full impact of PMTCT when implemented at scale at a national level. Some areas within countries may also have been left out in the retrieved studies.

As this review sought articles specifically addressing PMTCT and health services and systems, it may have missed publications reflecting recent increased attention and funding for health system strengthening through HIV programmes, which did not specifically address PMTCT. These papers may yield insights into broader health system challenges and promising approaches to integration of other HIV programmes, potentially including PMTCT.

## Conclusion

PMTCT improves maternal and child health through preventing the spread of HIV infection in SSA countries. There is evidence of a positive impact of PMTCT on primary care for mothers and children, beyond HIV. The provision of PMTCT services increases the availability, the accessibility and the utilisation of antenatal care and other health care services, especially when the intervention is linked to PMTCT programs as part of mainstream MCH services. However, this review also documented a large number of challenges involved both in implementing and in understanding the effects of PMTCT integration. Without robust information systems and rigorous and systematic research on the health system as a whole as well as on its various services and activities, evidence will continue to be fragmented and firm conclusions will continue to be impossible to draw.

While some vertical programs persist, PMTCT services are increasingly being integrated at different levels within routine health services and the health systems. Our study documented the challenges and weaknesses that face health care services and health systems in connection with PMTCT services. These range from structural, governance and resourcing challenges within and between countries, to weak information reporting systems and require more and better coordination and collaboration within and beyond HIV programs, directly related health services, communities, the plural health sector, and other sectors at national and global levels. These broad health system and social challenges cannot be solved by PMTCT interventions alone and there is a need of working together or collaborating with other sectors out of health system.

## Abbreviations

AIDS: Acquired Immune Deficiency Syndrome
ANC: Antenatal care
ART: Antiretroviral therapy
ARV: Antiretrovirals
CCWs: Community care workers
FP: Family planning
HIV: Human Immunodeficiency Virus
MCH: Maternal and child health
MTCT: The Mother-To-Child Transmission of HIV
NGOs: Non-Governmental Organisations
PMTCT: Prevention of mother-to-child transmission of HIV
PNC: Postnatal care
RSA: The Republic of South Africa
SRH: Sexual and reproductive health
SSA: Sub-Saharan Africa
STI: Sexually Transmitted Infection
TB: Tuberculosis
UNAIDS: The United Nations Programme on HIV and AIDS
UNFPA: The United Nations Population Fund
UNGASS: The United Nations General Assembly Special Session
UNICEF: The United Nations Children’s Fund
WHO: World Health Organisation
